# Establishing a TaqMan-Based Real-Time PCR Assay for the rapid detection and quantification of the newly emerged duck tembusu virus

**DOI:** 10.1186/1743-422X-8-464

**Published:** 2011-10-07

**Authors:** Liping Yan, Pixi Yan, Jiewen Zhou, Qiaoyang Teng, Zejun Li

**Affiliations:** 1 Division of Avian Infectious Diseases, Key Laboratory of Animal Parasitology of the Ministry of Agriculture, Shanghai Veterinary Research Institute, Chinese Academy of Agricultural Sciences, 518 Ziyue Road, Minhang District, Shanghai 200241, China

## Abstract

To establish an accurate, rapid, and a quantifiable method for the detection of the newly emerged duck Tembusu virus (DTMUV) that recently caused a widespread infectious disease in ducks in China, we developed a TaqMan-based real-time PCR assay by using E gene-specific primers and a TaqMan probe. This real-time PCR assay was 100 times more sensitive than the conventional PCR. The reproducibility and specificity of the real-time PCR assay were confirmed using plasmids containing E genes or RNAs and DNAs extracted from well-known viruses causing duck diseases. The reliability of this real-time PCR assay was confirmed in 19 of the 24 swab samples, 22 of the 24 tissue samples collected from experimentally infected ducks, as well as 15 of the 21 clinical samples collected from sick ducks since they were verified as DTMUV-positive. The results reveal that the newly established real-time PCR assay might be a useful diagnostic method for epidemiologically investigating and closely observing the newly emerged DTMUV.

## Introduction

In April 2010, an outbreak of the newly emerged duck Tembusu virus (DTMUV) caused a range of symptoms in infected ducks, including retarded growth, high fever, loss of appetite, decreased egg production, and death. The outbreak was first detected in southeast China, including the Shanghai, Zhejiang, Jiangsu, Fujian, and Anhui provinces and continued until the winter [1-3]. Shelducks in the provinces of Shandong, Henan, Hunan, Hubei, and Jiangxi were under a huge threat by this newly emerged virus with 100% infection and morbidity rates and mortality rates from 5% to 30%. To date, more than 10 million shelducks have been infected, and approximately 1 million have died. However, being a novel emerged causative agent in ducks, the lack of fast, easy, and reliable methods to diagnose the disease has limited researchers' abilities to conduct epidemiological investigations and monitor DTMUV. In this paper, we described a TaqMan-based real-time PCR assay for this newly emerged virus.

## Materials and methods

### Primers and probe design

Specific primers and a TaqMan probe to detect DTMUV were designed using Primer Express 2.0. The target sequence (115-bp long) was selected from a relatively conserved region on the E gene of DTMUV, Fengxian virus (FXV) strain (GenBank accession no. HQ833330). The fluorescent reporter dye, FAM, was located at the 5' end of the probe; the quencher, TAMRA, was located at the 3' end. The primers used were the EF primer (forward, 5'-TGTCTTATGCAGGTACCGATG-3') and the ER primer (reverse, 5'-CGTATGGGTTGACTGTTATCA-3'). The probe used was the TaqMan probe EP (FAM-AGTTCCCATATCCATGTC-TAMRA).

### Viruses

FXV, one of the DTMUV isolates, was propagated in specific pathogen-free embryonated chicken eggs. The embryos were carefully monitored, and those that died 24-120 h post-inoculation were pooled, homogenized, and centrifuged along with the chorioallantoic membranes. The supernatants were stored at -80°C until they were used for molecular analyses. The FXV titer was determined according to the 50% egg lethal dose (ELD_50_).

### RNA extraction and reverse transcription

RNA was extracted from FXV by using the RNeasy mini kit (Qiagen Inc., Valencia, CA) according to the manufacturer's instructions. The total RNA was stored at -80°C until use.

First-strand cDNA was synthesized using AMV reverse transcriptase (TaKaRa Biotechnology, Dalian, China) according to the manufacturer's instructions. Briefly, the following reagents were added and mixed: 4 μL of 5× reverse transcriptase buffer, 2 μL of dNTP mixture (10 mmol/L), 1 μL of random primer (50 mmol/L), 2 μL of AMV reverse transcriptase, 0.5 μL of RNAase inhibitor (40 U/μL), 5 μL of total RNA, and 5.5 μL of RNAase-free water. The reaction mixture was sequentially incubated at 42°C for 60 min and then at 72°C for 15 min. The DNA was subsequently stored at -20°C.

### Preparing the standard plasmid DNA

PCR was used to amplify the E gene in a reaction mixture consisting of 5 μL of 10× buffer, 4 μL of dNTP, 2 μL of each primer (10 μM of EF and ER), and 0.5 μL of Ex Taq Hot Start (TaKaRa Biotechnology, Dalian, China). The thermal conditions were as follows: initial denaturation at 94°C for 5 min; 35 cycles at 94°C for 30 s, 55°C for 30 s, and 72°C for 30 s; and a final extension at 72°C for 10 min.

The PCR product was then inserted into a pMD18-T vector, (TaKaRa Biotechnology, Dalian, China). After the recombinant plasmid was amplified in DH5α host bacteria, it was purified using a commercial test kit (Axygen Scientific Inc., Union, CA). The concentration of the plasmid was determined according to the OD_260 _value, and the number of copies of the plasmid was calculated [4]. The plasmids were maintained at -20°C for use as standard DNA in subsequent experiments.

### Establishing a standard curve for the real-time PCR

The real-time PCR for the E gene was conducted in a 25 μL reaction system containing the following ingredients: 2.5 μL of 10× buffer, 2.5 μL of dNTP, 1 μL of each primer (10 μM of EF and ER), 0.6 μL of the probe (10 μM of EP), 1 μL of recombinant plasmid, 0.2 μL of Ex Taq Hot Start, and 16.2 μL of sterile water.

Real-time PCR was performed on Mastercycler ep realplex (Eppendorf, Germany) by using the following thermal cycles: 95°C for 2 min, 40 cycles at 95°C for 20 s, and 54°C for 1 min. The assay was repeated at least 3 times with each template and the negative control.

### Determining the specificity of the real-time PCR

To determine the specificity of the real-time PCR, the FXV and 5 well-known viruses causing infectious diseases in ducks, including duck plague virus (DPV), avian influenza virus (AIV), duck hepatitis virus (DHV), duck parvovirus (DPVV), and Newcastle disease virus (NDV), were tested under the conditions described above.

### Sensitivity of the real-time PCR

To determine the lower detection limit, the plasmid stock was diluted with sterile water to 5 × 10^7 ^copies/μl, 5 × 10^6 ^copies/μl, 5 × 10^5 ^copies/μl, 5 × 10^4 ^copies/μl, 5 × 10^3 ^copies/μl, 5 × 10^2 ^copies/μl, 5 × 10^1 ^copies/μl, and 5 × 10^0 ^copies/μl. Four replicates of each dilution and 2 negative controls (blanks) were then tested by the real-time PCR and conventional PCR.

### Reproducibility of the real-time PCR

To test the reproducibility of the real-time PCR, standard plasmids in 3 different concentrations (5 × 10^5 ^copies/μl, 5 × 10^4 ^copies/μl, and 5 × 10^3 ^copies/μL) were aliquoted and stored at -80°C for future use as templates for intra- and inter-assay comparisons. The variations in the inter-assay were assessed by testing 5 replicates of each concentration in a single round of real-time PCR, and the variations in the intra-assay were assessed by repeating 5 rounds of real-time PCR. The coefficients of variation (CV) for the *C*t values of the intra- and inter-assay comparisons were determined.

### Detecting samples from experimentally or naturally infected ducks

The shelducks were inoculated intranasally with 10^5 ^ELD_50 _of the virus in 0.2 mL of supernatant of homogenized embryos and chorio-allantoic membranes of eggs that died 24-120 h post-inoculation with FX2010. One day after they were inoculated, 2 contact ducks were introduced into the isolator and placed among the ducks that had been inoculated intranasally. Oropharyngeal and cloacal swabs were collected from all ducks on days 2, 3, and 5 post-inoculation. All swabs were suspended in 1 mL of phosphate-buffered saline (PBS) and used for RNA extraction with an RNeasy Mini kit (Qiagen) or virus isolation. Two ducks from the inoculated group were euthanized 4 and 7 days post-inoculation, respectively. Two contact ducks were euthanized 6 days post-contact, and 2 non-infected ducks were euthanized as negative controls. Samples of blood, liver, spleen, kidneys, heart, brain, pancreas, and lungs were collected from the euthanized ducks. The samples were weighed, homogenized, and diluted in PBS (pH 7.4) at a ratio of 1:1 (mL/g). A portion of each sample was used for total RNA extraction and applied to the reverse transcriptase real-time PCR assay. In addition, a portion of each sample was used to isolate the virus in embryonated chicken eggs.

Twenty-one clinical samples, including blood, spleen, liver, and brain samples, from sick farm-raised ducks suspected of being infected with DTMUV were tested using both real-time PCR and conventional PCR.

## Results

### Establishing a standard DNA template

The concentration of the original extract of the recombinant plasmid containing the FXV E gene was 154.39 ng/μL. Accordingly, the extract contained 5.00 × 10^10 ^plasmids/μL.

### Establishing a standard curve for the real-time PCR

**Figure 1:**
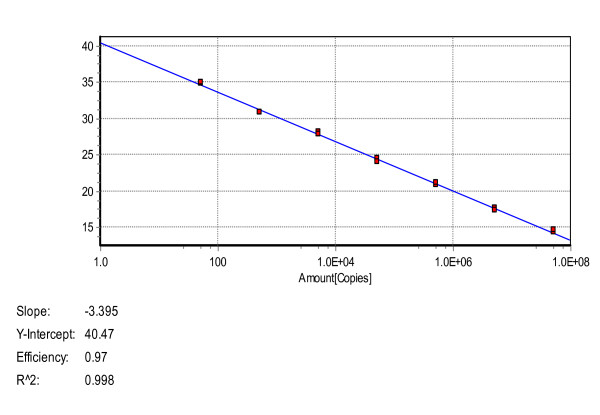
**Standard curve for the real-time PCR**. The X-axis represents copies of the plasmids, and the Y-axis represents the cycle threshold (*C*t). The assays were linear from 10^7 ^to 10^1 ^template copies; R^2 ^was 0.998, and the reaction efficiency was 97% for the E gene.

The standard curve was generated using different template concentrations ranging from 5.00 × 10^7 ^to 5.00 × 10^1 ^copies/μL obtained by 10-fold serial dilutions. The assays were linear with R^2 ^values of 0.998; the reaction efficiency for the E gene was 97% (Figure 1).

### Real-time PCR specificity

**Figure 2:**
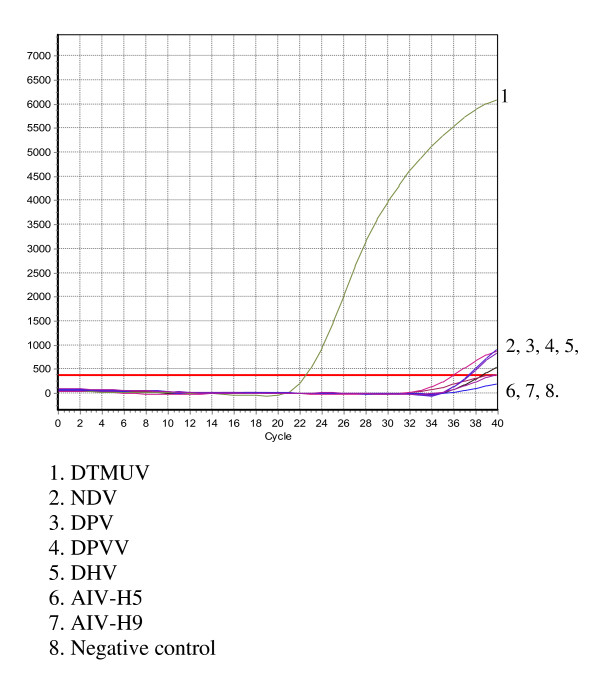
**Real-time PCR specificity**. The X-axis represents cycles, and the Y-axis represents the fluorescence data.

The FXV and 5 different duck-causative agents of duck infectious diseases were tested using the real-time PCR. FXV tested positive, while the other virus samples (i.e., DPV, AIV, DHV, DPVV, and NDV) and the water control tested negative (Figure 2).

### Real-time PCR sensitivity

**Figure 3:**
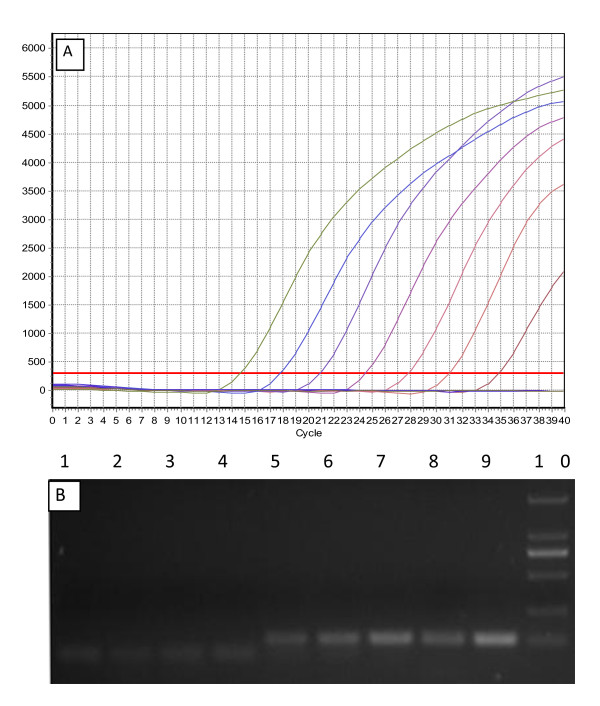
**Comparison of the sensitivity of the real-time and conventional PCR**. A. The sensitivity of the real-time PCR assay with the 10-fold dilution series of the standard plasmid DNA from 5 × 10^7 ^to 5 × 10^0 ^copies. The X-axis represents cycles, and the Y-axis represents the fluorescence data. The detection limit was 50 copies. B. Electrophoresis analysis of conventional PCR with EF and ER primers. Lane 1: H_2_O (control). Lanes 2-9: Amplification results of 5 × 10^0 ^to 5 × 10^7 ^template copies. Lane 10: DNA Marker DL2 000.

The detection limit of the real-time PCR was 50 copies, while the conventional PCR assay showed positive results only when more than 5 × 10^3 ^copies of the template were used (Figure 3B). Therefore, the sensitivity of the real-time PCR is about 100 times greater than that of conventional PCR.

### Reproducibility of the real-time PCR

When the 3 different concentrations of plasmids were subjected to the real-time PCR assay, the intra- and inter-assay CV values (i.e., the means from each of 5 replicates) were relatively small (Table 1).

**Table 1:** Intra- and inter-assay reproducibility of the real-time PCR

Standard plasmids	Copies	Intra-assay (*C*T Value)			Inter-assay (*C*T value)		
		Mean	SD	CV(%)	Mean	SD	CV(%)
1	5.0 × 10^3^	28.09	0.25	0.89%	28.00	0.20	0.72%
2	5.0 × 10^4^	24.56	0.15	0.61%	24.68	0.14	0.57%
3	5.0 × 10^5^	21.74	0.09	0.42%	21.90	0.21	0.97%

### Detecting DTMUV in the samples from artificially and naturally infected ducks

Twenty-four swab samples were collected from the ducks inoculated intranasally with FXV and those in contact with infected ducks. When the swabs were tested by the real-time PCR, 19 of them were DTMUV-positive; 7 of these were positive according to virus isolation (Tables 2 and 3).

**Table 2:** Virus detection from oropharyngeal and cloacal swabs of the experimentally infected ducks by real-time PCR and virus isolation

Samples	Days PI ^c^	Oropharyngeal swabs			Cloacal swabs		
		
		Real-time PCR		Virus isolation	Real-time PCR		Virus isolation
					
		*C*t^a^	Result^b^		*C*t^a^	Result^b^	
1	2	> 35	-	-	> 35	-	-
2	2	33.67 ± 2.60	+	-	33.58 ± 0.61	+	+
3	3	33.59 ± 0.05	+	-	32.79 ± 0.10	+	-
4	3	28.21 ± 0.09	+	+	28.37 ± 0.19	+	-
5	5	32.06 ± 0.29	+	+	27.34 ± 0.12	+	-
6	5	31.10 ± 0.33	+	-	25.46 ± 0.22	+	+

(+): positive; (-): negative

^a ^*C*t values obtained for the experiment are expressed as mean ± standard deviation (n = 3) if greater than zero.

^b ^*C*t < 35 was considered positive (+).

^c ^PI: post-inoculation.

**Table 3:** Virus detection from oropharyngeal and cloacal swabs of the contacted ducks by real-time PCR and virus isolation

Samples	Days PC^c^	Oropharyngeal swabs			Cloacal swabs		
		
		*C*t^a^	Result^b^	Virus isolation	*C*t^a^	Result^b^	Virus isolation
1	1	> 35	-	-	> 35	-	-
2	1	32.48 ± 0.84	+	-	> 35	-	-
3	2	33.14 ± 0.57	+	-	33.30 ± 0.38	+	+
4	2	29.87 ± 0.10	+	-	32.11 ± 0.74	+	-
5	4	30.32 ± 0.12	+	-	29.92 ± 0.04	+	+
6	4	30.93 ± 0.19	+	+	27.34 ± 0.12	+	-

+, -, ^a^, and ^b ^have the same meanings as in Table 2.

^c ^PC: post-contact.

Regarding blood and tissue samples, the positivity rate of all samples by the real-time PCR was obviously higher than by virus isolation. Specific RNAs of the DTMUV E gene were detected in all liver and kidney samples by the real-time PCR; meanwhile, only 2 of the 4 liver samples and 3 of the 4 kidney samples were DTMUV-positive by virus isolation (Table 4). More than 10^4 ^copies of specific DTMUV RNA were detected in all samples determined to be DTMUV-positive by virus isolation. In addition, the samples from non-infected ducks produced negative results when tested by both real-time PCR and virus isolation.

**Table 4:** Quantification of DTMUV in tissue samples by real-time PCR^a ^and virus isolation^b ^

Samples	Duck 1		Duck 2		Duck 3		Duck 4	
	Real-time PCR	Virus isolation^b^	Real-time PCR	Virus isolation^b^	Real-time PCR	Virus isolation^b^	Real-time PCR	Virus isolation^b^
Blood	6.56 ± 0.06	2.50	4.46 ± 0.36	0.98	-	-	6.80 ± 0.03	1.25
Liver	4.88 ± 0.03	-	4.53 ± 0.08	-	5.54 ± 0.05	0.97	6.66 ± 0.02	2.17
Lungs	5.49 ± 0.04	2.75	5.02 ± 0.01	1.38	6.11 ± 0.02	3.25	6.48 ± 0.01	3.50
Kidneys	5.01 ± 0.02	1.75	4.02 ± 0.06	-	5.49 ± 0.15	3.50	5.42 ± 0.05	2.75
Brain	-		5.04 ± 0.06	1.75	5.43 ± 0.01	2.50	6.50 ± 0.08	2.25
Pancreas	6.75 ± 0.04	2.25	6.91 ± 0.01	2.50	7.52 ± 0.02	1.67	7.56 ± 0.02	3.25

(-): Negative results with the real-time PCR or virus isolation assay.

Ducks 1 and 2 were inoculated intranasally with 10^5 ^ELD_50 _FXV and euthanized 4 and 7 days post-inoculation, respectively.

Ducks 3 and 4 were introduced into the isolator, placed with the intranasally inoculated ducks, and euthanized 6 days post-contact.

^a ^Unit: log 10 copies/mL for blood and log 10 copies/g for other tissue samples.

^b ^Unit: log 10 ELD_50_/mL for blood and log 10 ELD_50_/g for other tissue samples.

To determine the causative agents of disease in farm-raised ducks, 21 clinical samples collected from sick ducks were tested by real-time PCR and conventional PCR. Of these, real-time PCR showed that 15 were DTMUV-positive, while conventional PCR showed that only 11 were DTMUV-positive.

## Discussion

Many methods such as histological examination, serological testing, virus isolation, and molecular assays are available for the diagnosis of flavivirus infections. Virus isolation is time consuming since it often requires 3-4 days. Histological examination and serological testing require specific antibodies to avoid serological cross-reactivity between different viruses [5,6]. Because DTMUV is a newly emerged pathogen, developing a specific antibody against it will take time. To rapidly diagnose the infectious disease caused by DTMUV, molecular diagnostic methods such as reverse transcriptase PCR, reverse transcriptase real-time PCR, and loop-mediated isothermal amplification--all of which have been successfully used to diagnose other flaviviruses [6-11]--are useful for the epidemiological surveillance of DTMUV in China.

This paper describes a novel reverse transcriptase real-time PCR assay for DTMUV. The primers and probe were selected from the E gene of the DTMUV genome because this region is relatively conserved among DTMUV isolates. When the FXV and 5 other duck viruses were tested using real-time PCR, only the FXV tested positive. The results indicate that the real-time PCR assay is specific to DTMUV. In addition, the real-time PCR assay, including RNA extraction, took approximately 3.5 h, which is several hours or even days faster than conventional PCR and other virus isolation methods. Besides producing rapid results, reverse transcriptase real-time PCR has the advantages of sensitivity and efficiency for large-scale screening and virus quantification compared to conventional reverse transcriptase PCR [12-16]. Our newly established real-time PCR assay had a detection limit of 50 copies for DTMUV, which is 100 times more sensitive than conventional reverse transcriptase PCR. The method described here is sufficiently sensitive and specific for the diagnosis of DTMUV in both artificially and naturally infected ducks.

## Conclusions

We described an easy, fast, and a reliable diagnostic method that can detect and quantify newly emerged DTMUV by using a reverse transcriptase real-time PCR assay.

## Abbreviations

AIV: avian influenza virus; *C*t: cycle threshold; CV: Coefficients of variation; DHV: duck hepatitis virus; DPV: duck plague virus; DPVV: duck parvovirus; DTMUV: duck Tembusu virus; FXV: Fengxian virus; NDV: Newcastle disease virus; PBS: phosphate-buffered saline; PC: post-contact; PI: post-inoculation

## Competing interests

The authors declare that they have no competing interests.

## Authors' contributions

ZJL designed the probe and primers and revised the manuscript. LPY wrote the manuscript and carried out the experiments with the help of PXY who carried out virus isolation, JWZ who extracted RNA from the samples, and QYT who participated in the sequence alignment. All the authors have read and approved the final manuscript.
